# Temperature synchronization of the *Drosophila* circadian clock protein PERIOD is controlled by the TRPA channel PYREXIA

**DOI:** 10.1038/s42003-019-0497-0

**Published:** 2019-07-01

**Authors:** Sanne Roessingh, Mechthild Rosing, Martina Marunova, Maite Ogueta, Rebekah George, Angelique Lamaze, Ralf Stanewsky

**Affiliations:** 1 Department of Cell and Developmental Biology, University College London, London, WC1E 6DE UK; 2 Institute for Neuro and Behavioral Biology, Westfälische Wilhelms University, Münster, D-48149 Germany

**Keywords:** Circadian rhythms and sleep, Ion channels in the nervous system

## Abstract

Circadian clocks are endogenous molecular oscillators that temporally organize behavioral activity thereby contributing to the fitness of organisms. To synchronize the fly circadian clock with the daily fluctuations of light and temperature, these environmental cues are sensed both via brain clock neurons, and by light and temperature sensors located in the peripheral nervous system. Here we demonstrate that the TRPA channel PYREXIA (PYX) is required for temperature synchronization of the key circadian clock protein PERIOD. We observe a molecular synchronization defect explaining the previously reported defects of *pyx* mutants in behavioral temperature synchronization. Surprisingly, surgical ablation of *pyx*-mutant antennae partially rescues behavioral synchronization, indicating that antennal temperature signals are modulated by PYX function to synchronize clock neurons in the brain. Our results suggest that PYX protects antennal neurons from faulty signaling that would otherwise interfere with temperature synchronization of the circadian clock neurons in the brain.

## Abstract (web summary)

Sanne Roessingh et al. report that the *Drosophila* TRPA channel encoded by *pyrexia* is required for temperature synchronization of PERIOD, a key circadian clock protein. They find that ablating the antennae of *pyx* mutants partially rescues their synchronization defect, suggesting that PYX functions in the antennae to buffer temperature signals to the brain.

## Introduction

Organisms use daily fluctuations of light and temperature to synchronize their internal circadian clocks with the external time. In ectothermic animals and in plants, daily temperature cycles are particularly suited for clock synchronization, but they also act as external time cues (or zeitgebers) for circadian clock resetting in mammals^1,2^. In *Drosophila*, temperature cycles are potent zeitgebers as day and night differences of as little as 2 °C are sufficient to synchronize molecular and behavioral rhythms^3,4^. Whereas 10-min light pulses robustly shift the clock^5^, temperature pulses must be outside the physiological range (e.g., 37 °C, see reference ^6^) or last longer than 6 h^7^ to be effective. This explains why fast responding thermoreceptors, regulating temperature preference or heat responses, do not mediate temperature synchronization of circadian clocks^8,9^. Instead clocks seem to rely on receptors that are able to integrate temperature information over long periods of time, ensuring that they are not reset by short and abrupt (e.g., weather-induced) temperature changes.

In order to reliably extract day/night information from slowly changing temperatures it appears that *Drosophila* recruits a special sensory system, the chordotonal organs (ChO), well described for mechanosensitive functions in proprioception and hearing^10,11^. *nocte* mutants have structural ChO defects and fail to properly synchronize their clocks to temperature cycles^12,13^. Moreover larval ChO are known to mediate temperature responses^14–16^ and we previously showed that the variant Ionotropic Receptor IR25a is expressed in ChO neurons, can sense small temperature differences, and is required for synchronization to shallow temperature cycles^3^. The thermo-sensitive TRPA channel PYREXIA (PYX) is also expressed in ChO, but in nonneuronal “cap cells,” connecting the dendritic cilia of the ChO neuron to the cuticle^17,18^. The PYX channel protects flies from rapid paralysis at 40 °C, and in heterologous cells opens in response to temperatures >40 °C^19^. Because PYX is primarily permeable for K^+^, its high activation temperature lead to the hypothesis that PYX protects neurons from overexcitation during high-temperature stress^19^. Surprisingly, we found that PYX is also required for temperature synchronization to 12 h 16 °C:12 h:20 °C, but not to warmer temperature cycles (e.g., 25 °C:29 °C, see reference^18^). Green et al.^20^ reported that under seminatural conditions of gradually changing light:dark (0–400 lx) and temperature (25 °C:35 °C) cycles, *pyx* mutants lacked the typical morning and evening activity peaks. Moreover, antennal PYX expression is required for the activation of TRPA1 expressing anterior cell neurons (AC) brain neurons at 27 °C^21^. In addition, PYX has an important temperature-independent function in geotactic behavior^17,22^. Taken together this indicates that PYX is a versatile channel able to detect temperature changes over a large range with additional temperature-independent roles.

Interestingly, PYX expressing neurons located in the second or third segment of the antenna project to AC neurons^21^. The third segment contains heat and cold sensing cells in the aristae and sacculus mediating temperature preference behavior^23^, but *pyx–gal4* expression in this segment does not map to these structures^21^. Moreover, hot and cold cells do not project to the anterior cell neurons but into separate zones of the proximal antennal protocerebrum (PAP)^23,24^. In the second segment, *pyx–gal4* is expressed in cap cells of Johnston’s Organ, a specialized ChO for sound, gravity, and wind detection^10,17^, whereas the identity of *pyx–gal4* expressing neurons in the second and third antennal segments remains unknown^21^. Because surgical removal of the antennae does not prevent circadian clock synchronization, antennal thermo sensors are not required for temperature resetting^3,13^.

In order to synchronize the circadian clock, temperature changes captured by peripheral sensors must reach the ~150 clock neurons in the brain. These clock neurons are defined by the expression of clock genes like *period* (*per*) and *timeless* (*tim*) and are arranged in locally distinct but interconnected groups [small and large ventral lateral neurons (s-LNv and l-LNv), dorsal lateral neurons (LNd), Dorsal Neurons (DN1, DN2, and DN3), and lateral posterior neurons (LPN)]^25^. Cell-autonomous transcriptional feedback loops involving *per*, *tim,* and other clock genes result in 24-h rhythms of accumulation and degradation of clock gene products within the clock neurons^25^. These endogenous oscillations are accompanied by daily fluctuations of clock neuronal activity, which support the molecular rhythms and serve as rhythmic synchronizing and output signals^26,27^. One important role of the clock neuronal circuit is integrating incoming environmental signals to produce synchronized molecular oscillations, which in turn regulate the daily behavioral activity rhythms of the fly. How temperature signals reach the brain clock neurons is not known, but *nocte* and *IR25a* mutants show temperature specific synchronization defects of TIM expression particularly in the DN and LNd subgroups^3,28^. This suggests a connection between ChO and subsets of the dorsally located clock neurons, which is also supported by ChO-(and *nocte-*) dependent clock synchronization through rhythmic vibrational signals^29^. Moreover, the DN1 are activated by cold and inhibited by warm temperatures and *nocte* function in the fly body as well as thermoreceptors in the terminal antennal segment, the aristae, contribute to these responses^30^. Recently, the variant ionotropic glutamate receptors IR21a, IR25a, and IR93a have been shown to mediate thermal responses to both cooling and heating in thermosensitive arista neurons, indicating their potential role in temperature synchronization^31^.

Here we show that PYX is required for normal synchronization of the molecular clock in different subsets of clock neurons. Strikingly, removal of the antennae in flies lacking PYX partially rescues behavioral synchronization suggesting that PYX normally gates temperature input to clock neurons. Consistent with this and its predicted role in protecting neurons from over excitation during heat stress^19^, lack of PYX specifically increases calcium levels during temperature cycles and artificial activation of PYX expressing neurons mimics the behavioral PYX loss-of-function phenotype.

## Results

### Removal of the antenna partially restores temperature synchronization of *pyx*^3^ mutant flies

**Fig. 1:**
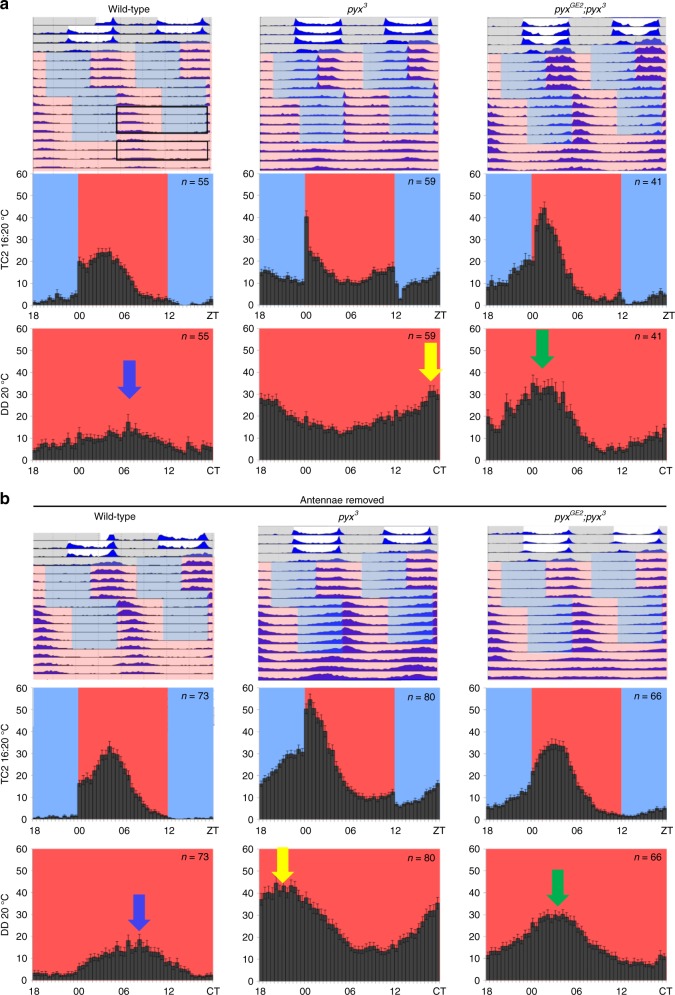
Ablation of the antennae partially restores temperature synchronization defects of *pyx*^*3*^ mutants. **a** Average locomotor activity of wild-type, *pyx*^*3*^, and *pyx*^*GE2*^*;pyx*^*3*^ male flies was measured during DDTC 20 °C:16 °C. Top row: representative double plotted actograms show average locomotor activity of indicated genotypes plotted in blue. Three days of light-dark cycles (at constant 20 °C) were followed by TC1 (5 days of 20 °C:16 °C in constant darkness), which was 6 h delayed compared with previous light-dark, followed by TC2 (5 days of 20 °C:16 °C in constant darkness), which was 7 h delayed compared with TC1, followed by 4 days of free-running conditions at constant 20 °C. Numbers of individuals in representative actograms range between *n* = 14 and *n* = 31. White and gray background color indicates lights-on and lights-off, and red and blue background colors indicate warm (20 °C) or cold (16 °C) temperature, respectively. Histograms show average locomotor activity during the last 3 days of TC2 (middle row) and the second and third day of free-running conditions (constant darkness 20 °C) (bottom row). Error bars indicate s.e.m. and *n* numbers are indicated. **b** Average locomotor activity of wild-type, *pyx*^*3*^ and *pyx*^*GE2*^*;pyx*^*3*^ male flies with ablated antennae was measured during and after DDTC 20 °C:16 °C as in **a**. Colored arrows point to main activity peak during constant conditions to aid comparison between intact and antennae-ablated flies

*pyx* is required for normal behavioral synchronization to 20 °C:16 °C temperature cycles, but the neuronal substrates of this phenotype are not known^18^. Since PYX and *pyx–gal4* are both expressed in the antennae^17,19^ (Supplementary Fig. 1) and these sensory organs play an important role in temperature sensing^21,23,24,30,31^, we hypothesized that *pyx* signaling from the antennae contributes to temperature synchronization. We therefore compared temperature synchronization ability of wild-type, *pyx*^3^, and *pyxGE2;pyx*^3^ flies (carrying a genomic *pyx* rescue construct in the *pyx*^*3*^ mutant background^19^) with intact antennae (Fig. 1a) to flies with surgically removed second and third antennal segments (Fig. 1b).

To allow comparison with the original study^18^, flies were subjected to 3 days of light-dark cycles (at constant 20 °C), followed by 5 days of 20 °C:16 °C temperature cycles in constant darkness (DDTC1) with temperature cycle 1 (TC1) delayed 6 h compared to previous light-dark, followed by 5 days of 20 °C:16 °C temperature cycles (DDTC2) with temperature cycle 2 (TC2) delayed 7 h compared to TC1, and followed by 4 days of constant darkness at a constant temperature (20 °C) (Fig. 1)^18^. Consistent with our previous study, in TC2 wild-type flies exhibited their main activity during the first half of the warm phase (~ZT3–ZT5, where ZT0 indicates the transition from 16 to 20 °C) (Fig. 1a). After release into free-running conditions, the phase of this activity peak is maintained (Fig. 1a: compare position of the activity peak in the histograms), indicating that the circadian clock has been synchronized with the temperature cycles (Fig. 1a)^18,32^. Flies lacking the PYX channel show a very different behavior. During TC2, *pyx*^3^ flies show a drastic activity increase coinciding with the warm transition, followed by a steady activity decrease until the middle of the warm phase, and an activity increase up to the cold transition, after which they abruptly decrease their activity (Fig. 1a). Moreover, an additional activity peak occurred in the middle of the cold phase, which is maintained in the subsequent free-running conditions (Fig. 1a). The behavior of *pyx*^*3*^ flies closely resembles that of our previous report^18^, and confirms that PYX is required for synchronization to temperature cycles. *pyx*^*3*^ mutants carrying a genomic rescue construct (*pyx*^*GE2*^*;pyx*^*3*^) increased their activity shortly after the switch to the warm phase but in contrast to the *pyx*^*3*^ mutants kept increasing their activity levels for several hours (until ~ZT2–ZT3), followed by a steady drop in activity levels (Fig. 1a) as in wild-type flies. Nevertheless, the rescue flies do show considerable activity during the cold phase, but in contrast to *pyx*^*3*^ flies this activity is mainly restricted to its second half and steadily increases up to the warm transition (Fig. 1a). Importantly, as in wild-type flies the phase of the main activity peak at the end of TC2 is maintained in constant conditions (compare position of the activity peaks in the histograms). The behavior of the rescue flies during TC2 and constant conditions is therefore similar to that of wild-type flies, indicating at least a partial rescue by the *pyx* construct^18^.

Wild-type flies without antennae show very similar behavior to flies with intact antennae (Fig. 1a, b: histograms are plotted on the same time scale to allow a direct comparison), consistent with earlier findings showing that the antennae are not required for synchronization to temperature cycles^3,13^. Surprisingly, and in contrast to flies with intact antennae, the activity levels of antenna-less *pyx*^*3*^ mutants during TC2 slowly increase during the cold phase reaching peak levels during the early warm phase, very similar to the rescue flies with intact antennae (compare plots of *pyx*^*3*^ in Fig. 1b with those of the rescue flies in Fig. 1a). The activity peak at the end of TC2 also dictates the initial phase of the activity peak in free-running conditions indicating that *pyx*^*3*^ flies without antennae can partially synchronize their behavior with temperature cycles (Fig. 1b). In *pyxGE2;pyx*^*3*^ flies, the activity peak during TC2 is shifted further toward the middle of the warm phase when antennae are removed, which makes the behavior almost identical to that of wild-type flies (Fig. 1b, compare to Fig. 1a). The gradual improvement of synchronization after removing the antennae from *pyx*^*3*^ mutant and rescue flies is also apparent when comparing the average activity phase during constant conditions. While there is no difference between the peaks of wild-type flies with or without antennae (blue arrows), peaks are maximally separated between wild-typewild-type and *pyx*^*3*^ mutants with intact antennae (compare blue and yellow arrow in Fig. 1a). Removal of the antennae shifts the peak of *pyx*^*3*^ flies toward that of wild-type flies (compare blue and yellow arrows in Fig. 1b with those in Fig. 1a). Likewise, the activity peak of rescue flies with intact antennae is already close to that of wild-type flies (compare blue and green arrows in Fig. 1a), and antennal removal shifts this peak even further toward wild-type (compare blue and green arrows in Fig. 1b with those in Fig. 1a).

**Fig. 2:**
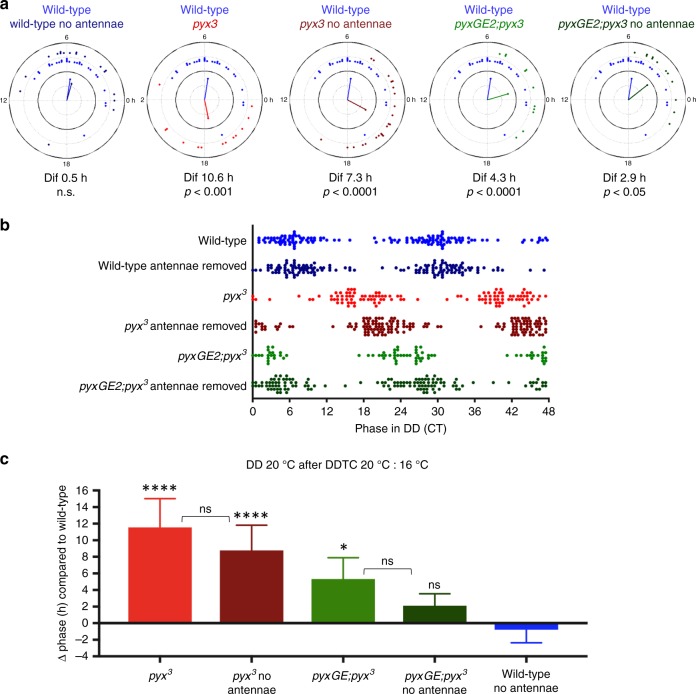
Quantification of activity peak phase in *pyx*^*3*^ and control flies with and without antennae. **a** Circular phase plots of a representative experiment showing the phase of peak activity during free-running conditions (constant darkness (DD) 20 °C, second and third day) TC2. Dots represent individual flies. The direction of the vector indicates mean phase of a genotype and the magnitude of the vector indicates the coherence of the group (variance around the mean of one genotype). For the plots shown here coherence was not significantly different between genotypes. Wild-type *n* = 28, phase = 5.4; wild-type no antennae *n* = 18, phase = 4.9; *pyx*^*3*^ *n* = 13, phase = −5.3; *pyx*^*3*^ no antennae *n* = 23, phase = −1.9; *pyx*^*GE2*^*;pyx*^*3*^ *n* = = 11, phase = 1.1; *pyx*^*GE2*^*;pyx*^*3*^ no antennae *n* = 12, phase = 2.5. The difference of peak activity to that of wild-type is given below the phase plots (Δ phase, (Dif) with associated *p* values). **b** Double plot of peak phase during free-running conditions (DD 20 °C, second and third day) after temperature cycles combining three experiments. Wild-type *n* = 70; wild-type no antennae *n* = 72; *pyx*^*3*^ *n* = 58; *pyx*^*3*^ no antennae *n* = 80; *pyx*^*GE2*^*;pyx*^*3*^ *n* = 38; *pyx*^*GE2*^*;pyx*^*3*^ no antennae *n* = 65. Sample size numbers are slightly less than in the histograms, because not for all flies that survived until the end of the experiment an activity peak could be determined. **c** Phase plots showing the difference of peak activity of the indicated genotypes compared to that of wild-type (Δ phase). Δ phase was determined on the second and third day of free run using circular statistics^33^ and the average of three independent experiments was plotted. For *n* numbers see **b**. One-way ANOVA followed by Tukey’s multiple comparisons test was applied to compare *pyx*^*3*^ and *pyx*^*GE2*^;*pyx*^*3*^ flies with and without antennae to wild-type without antennae (for comparison to wild-type with antennae see **a**) (****p < 0.0001, *p < 0.5). Using the same test *pyx*^*GE2*^*;pyx*^*3*^ flies with antennae were significantly different from *pyx*^*3*^ flies without antennae (*p* < 0.01), and *pyx*^*GE2*^;*pyx*^*3*^ without antennae were different from *pyx*^*3*^ flies both with and without antennae (*p* < 0.0001). Intragenotype phase differences of flies with or without antennae were not significant as indicated by brackets and in **a** for wild-type

To confirm that antennal removal improves temperature synchronization, we quantified both absolute phase and the phase difference between wild-type, *pyx*^*3*^ mutant, and rescue flies with and without antennae during the second and third day in constant conditions using circular phase plot analysis^33^ (Fig. 2a–c). Figure 2a shows a single representative experiment, where we compared the phase of flies with and without antennae to that of wild-type flies with intact antennae. As expected, there was no significant difference between wild-type flies with and without antennae. In contrast, the inability of *pyx*^*3*^ mutant flies to synchronize with temperature cycles resulted in a final phase difference with wild-type flies of 10.6 h. When the antennae were removed, synchronization ability was partially restored, and the final phase difference was reduced to 7.3 h. Introduction of a genomic rescue construct decreased the phase difference to 4.3 h and removing the antennae of these rescue flies further reduced the final phase difference with wild-type flies to 2.9 h (Fig. 2a). To better visualize the phase differences, we double-plotted the distribution of individual phase values from three independent experiments (Fig. 2b). Again, the phase of wild-type flies was not affected by the absence or presence of the antennae. The phase of *pyx*^*3*^ flies is almost opposite to that of wild-type, and removal of the antennae moved the phase distribution closer to that of wild-type (Fig. 2b). Finally, intact rescue flies show a phase distribution closer to that of wild-type compared to *pyx*^*3*^ flies without antennae, and removal of the antennae further shifts the distribution toward wild-type (Fig. 2b). The same tendency is also observed after comparing the average peak phases of three independent experiments with those of antennae-intact wild-type flies (Fig. 2c). From these behavioral results we conclude that PYX function within the antennae and/or other peripheral nervous system tissues must play a role in temperature synchronization of the circadian clock.

### PYX reduces intracellular Ca^2+^ reporter signals during temperature cycles

It has been proposed that PYX protects flies against high temperature stress by protecting neurons from inappropriate firing^19^. Therefore it seems plausible that removal of the channel could cause abnormal excitation of neurons also during temperature cycles. We hypothesize that lack of PYX causes faulty signaling from the antennae, which in turn interferes with temperature synchronization behavior in *pyx* mutant flies. When the antennae are removed, the interfering signal is reduced, resulting in partial restoration of synchronization (Fig. 1).

To test this idea we expressed the transcriptional reporter of intracellular Ca^2+^ (UAS-TRIC) in *pyx* expressing cells (*pyx–gal4*) in combination with bioluminescence-based luciferase reporting (*lexA-op-luc*)^34,35^. To verify that *pyx–gal4* is expressed in cells involved in temperature synchronization, we first used the *pyx–gal* driver to express *UAS-pyx-RNAi* (*pyx* > *pyx-RNAi*) and tested the behavior of these flies during 20 °C:16 °C temperature cycles. The *pyx* *>* *pyx-RNAi* flies showed behavior very similar to homozygous *pyx*^*3*^ flies, indicating RNAi-mediated *pyx* knockdown is directed to the relevant cells with *pyx–gal4* (Supplementary Figs. 2 and 3). The same mutant phenotype was observed by driving *pyx-RNAi* ubiquitously (*actin* > *pyx-RNAi*), whereas *pyx* knockdown in all clock cells (*timeless* *>* *pyx-RNAi*) resulted in nearly normal synchronization to temperature cycle (Supplementary Figs. 2 and 3).

**Fig. 3:**
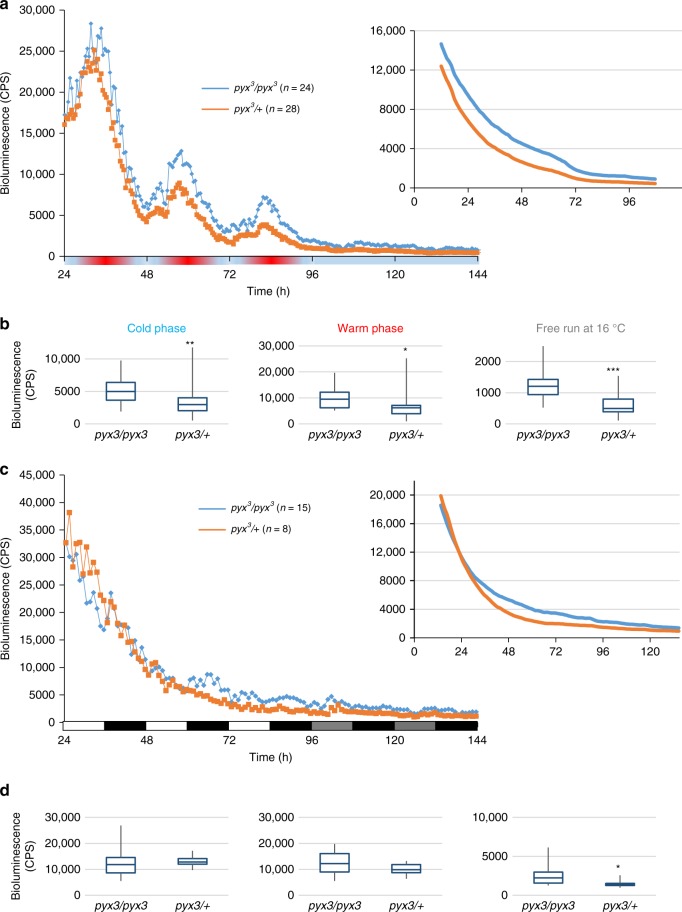
Loss of PYX causes higher intracellular Ca^2+^ levels during temperature cycles. **a** Bioluminescence recordings of male flies (*pyx*^*3*^/+ and *pyx*^*3*^*/pyx*^*3*^) expressing the TRIC Ca^2+^ reporter under the control of *pyx–gal4* and the firefly luciferase gene under control of the *lexA* operator (*lexA-op-luc*). High intracellular Ca^2+^ levels result in reconstitution of functional LexA transcription factor (TRIC) leading to increased *luciferase* expression and bioluminescence. Flies were exposed to ramped 16 °C:25 °C temperature cycle for 3 days, followed by 2 days at constant 16 °C and kept in constant darkness throughout the experiment. Left panel: bioluminescence (counts per second, CPS) from individual flies kept on luciferin containing food was measured every 30 min using a TopCount plate reader as previously described^60^. Inset on the right: trend plotted using ChronoStar to visualize differences in absolute levels between genotypes. Average traces from two experiments are shown. **b** Box plots of the average bioluminescence level distribution during cold, warm, and free-run phase were calculated and plotted using Excel. Middle lines indicate the median, bottom, and top borders, the first and third quartile, respectively. Whiskers indicate maximum and minimum values. Significance of differences between genotypes was determined by *t* test (*p < 0.05, **p < 0.005, ***p < 0.0005). **c** Bioluminescence recordings of male flies of the same genotypes as in **a** during 3 days of light-dark and 2 days of DD at constant 25 °C (left panel). Inset on the right: trend plotted using ChronoStar to visualize differences in absolute levels between genotypes. **d** Box plots of the average bioluminescence level distribution during light-dark and DD were generated as in **b** and the means of the two genotypes were compared by Student's *t* test (*p < 0.05). See also Supplementary Figs. 2 and 3

After this validation we combined the *pyx–gal4* driver with *pyx*^*3*^ and *lexA-op-luc* and crossed these flies to either *UAS-TRIC* or *UAS-TRIC pyx*^*3*^ flies. Fly behavior is monitored in glass tubes sealed with a simple cotton plug only, ensuring that changing temperature conditions in the incubator are quickly transmitted to the animal, whereas during the luciferase experiments flies are sitting in a sealed microtiter plate. To restrict movement in the *z*-axis (which we know considerably affects the luciferase signal, due to variable distance between the fly and the photomultiplier tube) flies are additionally covered with a small plastic dome. Because abrupt temperature changes cause additional artifacts (large bioluminescence spikes at the temperature transitions) we also used ramping temperature cycles, where the temperature changes about 1 °C/h. These measures likely dampen the amplitude of the temperature cycle experienced by the fly and we therefore performed the luciferase experiments both at 25 °C:16 °C (Fig. 3a, b) and 20 °C:16 °C (Supplementary Fig. 4A) temperature cycles in constant darkness for 3 days, followed by 2 days at constant 16 °C. Bioluminescence was recorded every 30 min using a TopCount plate reader (PerkinElmer) as previously described (e.g., see reference^28^). We observed robust bioluminescence fluctuations in both control and *pyx*^*3*^ flies, with bioluminescence increase and decrease following the ambient temperature, and constitutive low levels during constant 16 °C (Fig. 3a). To see if these oscillations reflect endogenous Ca^2+^ fluctuations within *pyx*-expressing cells, or simply a temperature response of the reporter system, we directly expressed *lexA* and *lexA-op-luc* (i.e., without the TRIC Ca^2+^ sensor) in non-*pyx* cells and observed the same high-amplitude bioluminescence rhythms (Supplementary Fig. 4B). Since luciferase activity does not change drastically with temperature (e.g., see reference^12^), this suggests that LexA activity is temperature dependent explaining the robust bioluminescence oscillations observed during temperature cycles.

We noted that the overall bioluminescence levels in *pyx*^*3*^ flies were higher compared to the controls, both during the warm and cold phase, as well as at constant 16 °C (Fig. 3a and Supplementary Fig. 4A). To better visualize the difference between mutant and wild-type, we calculated the moving average for 24 h windows separated by the 30 min sampling time using ChronoStar^37^. This analysis eliminates any rhythmic components in the data thereby revealing basic trends (in this case, a downward trend in both genotypes, caused by substrate depletion, calcium decrease, or a combination of both), and revealing higher TRIC-reported Ca^2+^ levels in the *pyx*^*3*^ mutant background at all times (Fig. 3a, inset). Comparison of the average reporter signals during the different conditions of the experiment shows that the differences between the mutant and the control are significant at all times for the 16 °C:25 °C regime (Fig. 3b). In the 16 °C:20 °C regime, the same trend was observed, but TRIC-reported Ca^2+^ levels were significantly increased only during constant conditions (Supplementary Fig. 4A). These results suggest that *pyx*-expressing cells contain more Ca^2+^ in the absence of the PYX channel, reminiscent of the proposed role for PYX in protecting neurons from over excitation^19^. To test the temperature dependency of this effect, we performed the same TRIC experiment exposing the flies to constant temperatures (25 °C) and light-dark cycles for 3 days, followed by 2 days in constant darkness. Strikingly, during light-dark cycles the *pyx*^*3*^ flies did not exhibit higher bioluminescence levels compared to controls (Fig. 3c, d). Although Ca^2+^ levels in the mutant were marginally increased in the subsequent constant darkness, the results indicate that the increased Ca^2+^ levels caused by the lack of PYX are specific for temperature cycles and do not occur during light-dark cycles.

### Constitutive activation of PYX expressing cells interferes with temperature synchronization

**Fig. 4:**
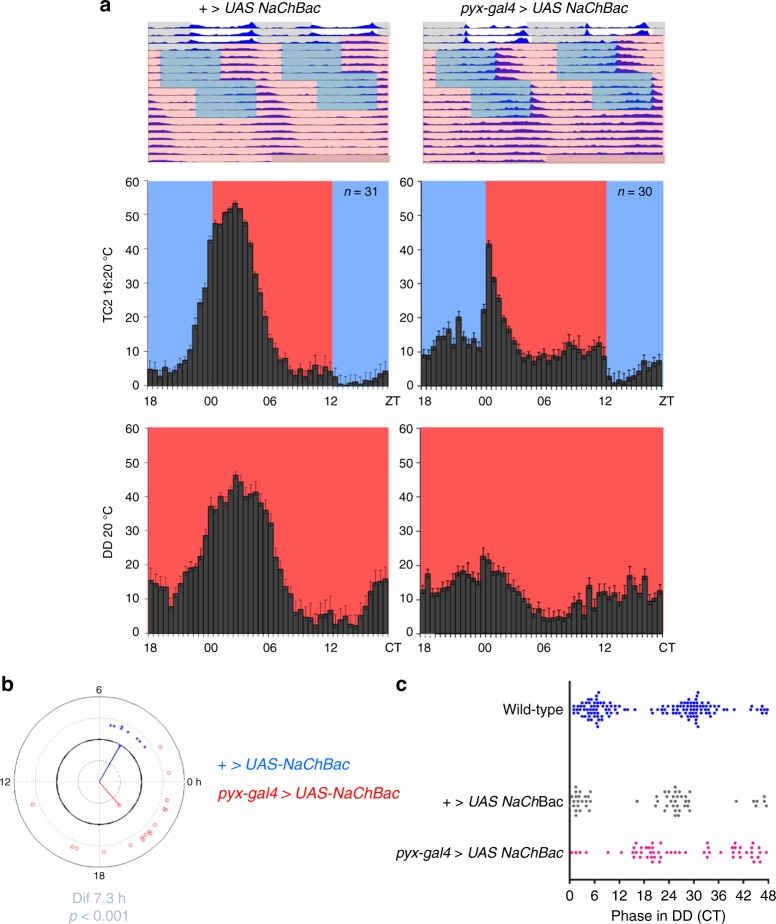
Constitutive activation of PYX neurons interferes with temperature synchronization. **a** Average locomotor activity of control (+ *>UAS-NaChBac*) and male *pyx–gal4* *>* *UAS-NaChBac* flies. Top row: representative double plotted actograms show average locomotor activity of indicated genotypes plotted in blue. Three days of light-dark cycles (at constant 20 °C) were followed by two shifted 20 °C:16 °C temperature cycles as described and plotted as in Fig. 1a. **b** Circular phase plot showing the phase of the behavioral peak during free-running conditions (constant darkness (DD) 20 °C) after DDTC 20 °C:16 °C (second and third day). Dots represent individual flies. The direction of the vector indicates mean phase of a genotype and the magnitude of the vector indicates the coherence of the group (variance around the mean). The difference (Dif) in mean phase between genotypes is given below the phase plots. Circular statistics were used to determine if phase differences were significant (as in reference^33^). For the plots shown here the coherence (variance around the mean of one genotype) was not significantly different between genotypes. Blue: *UAS-NaChBac/*+ *n* = 8, phase = 4.0; red, *pyx–gal4/UAS-NaChBac* *n* = 17, phase = −3.3. Δphase 7.3 h (*p* < 0.001). In an independent experiment we observed similar differences: *UAS-NaChBac/*+ *n* = 8, phase = 3.0; *pyx–gal4/UAS-NaChBac* *n* = 13, phase = −5.4. Δphase 8.4 h (*p* < 0.05). **c** Double plot showing absolute phase distribution of *UAS-NaChBac/*+ (*n* = 31) and *pyx–gal4/UAS-NaChBac* (*n* = 30) compared to wild-type (replotted from Fig. 2b)

If PYX protects neurons from over excitation during temperature cycles, constitutive activation of *pyx* neurons should block temperature synchronization, similar to the effects of *pyx*^*3*^. To test this, we activated *pyx* neurons by expression of the bacterial sodium channel NaChBac^38^. Strikingly, when exposed to shifted 16 °C:20 °C temperature cycles, flies exhibited a behavioral phenotype resembling that of *pyx*^*3*^ (Fig. 4a, compare to Fig. 1a). Flies with constantly activated *pyx* neurons showed an abrupt activity increase after the warm transition and an additional activity peak during the cold phase, both typical for *pyx*^*3*^ mutants (compare Fig. 4a with Fig. 1a). During the subsequent constant conditions (constant darkness, 20 °C) *pyx-gal4/UAS-NaChBac* mutants exhibit an 7.3 h phase difference of their average activity peak compared to controls (*p* < 0.001), very similar to *pyx*^*3*^ mutants (Fig. 4b, compare to Fig. 2a). This phase difference is also reflected in the distribution of absolute phase values, showing that most flies with constantly activated *pyx* neurons exhibit an almost opposite phase compared to control and wild-type flies (Fig. 4c). Under the same conditions *pyx–gal4/*+ and *UAS-NaChBac*/+ behave similar to wild-type flies, exhibiting their main activity peak during the first half of the warm phase and no peak during the cold phase (Fig. 4a and Supplementary Fig. 2, compare to Fig. 1a). These results suggest that the PYX channel indeed protects neurons from overexcitation during temperature cycles thereby contributing to correct temperature synchronization of the circadian clock neurons.

### PYX impacts PER expression in s-LNv and DN1 clock neurons during temperature cycles

**Fig. 5:**
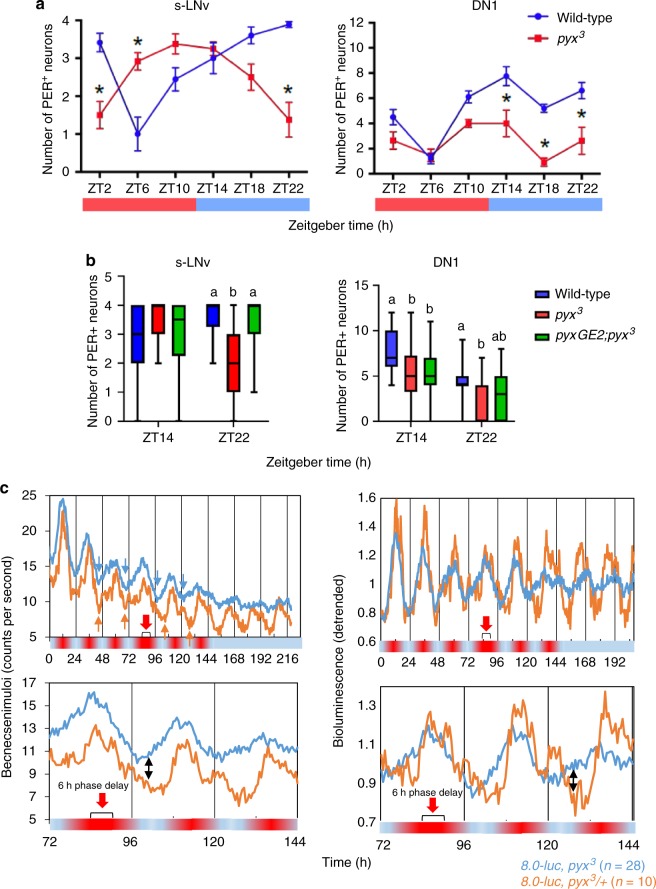
PYX is required for temperature synchronization of PER expression in subsets of the circadian clock neurons. **a** Average number of s-LNv and DN1 PER^+^ neurons detected at six time points during the last day of TC2 (16 °C:20 °C) (indicated in Supplementary Fig. 4A by red arrowheads). Error bars represent s.e.m. Sample size varies between six and eighteen hemispheres per time point. 20 °C (red) and 16 °C (blue) temperature cycle is indicated with the bar below each graph. Significant differences in the number of PER^+^ neurons between genotypes were determined using two-way ANOVA followed by Sidak’s multiple comparisons test in the case of significant interaction or significant effect of genotype (*p <  0.05). Costaining with PDF antibody allowed identification of s-LNv, whereas DN1 were identified by position within the brain relative to the PDF-labeled s-LNv projections. **b** Independent experiment analyzing number of PER^+^ neurons at ZT14 and ZT22 on the final day of TC2 and also including *pyxGE2, pyx*^3^ rescue flies. Box plots indicate median, first and third quartile, as well as maximum and minimum values. Sample size numbers range between eighteen and twenty eight hemispheres per time point. Significant differences in the number of PER^+^ neurons between genotypes were determined using two-way ANOVA followed by Tukey’s multiple comparisons test in the case of significant interaction or significant effect of genotype (different letter means *p* < 0.05 e.g. ‘a’’ vs. ‘b’’, shared letter means n.s. e.g. a vs. ab) (there was a significant genotype × time point interaction for s-LNv; there was no significant interaction but a significant effect of genotype alone for DN1). See Supplementary Fig. 4B, C for analysis of other clock neuron subsets. **c** Bioluminescence recordings of *pyx*^*3*^ and *pyx*^*3*^/+ flies carrying the *8.0-luc* PER-LUC reporter. Flies were exposed to ramped 16 °C:20 °C temperature cycle and bioluminescence was recorded every 30 min as described for Fig. 3. Delay of the temperature cycle is indicated by brackets and red arrows. Raw data are shown to the left, and detrended data to the right (see Methods). Orange and blue arrows point to the trough of expression before and after the temperature cycle shift for controls and *pyx*^*3*^ mutants, respectively. Day of the temperature cycle-shift and two subsequent days are shown in the bottom row. Double arrows point to phase difference between mutants and controls after the temperature cycle-shift

Because PYX is expressed exclusively within the peripheral nervous system, but synchronized behavior is driven by central nervous system clock neurons, we predicted that the lack of PYX interferes with signaling from the peripheral nervous system to the circadian clock neurons in the brain. To test this hypothesis we asked if PER cycling in clock neurons was altered in *pyx* mutant flies. Behavior of wild-type and *pyx*^*3*^ mutant flies was analyzed on day 4 of TC2 and individuals that displayed clear wild-type or mutant behavior, respectively, were collected for immunocytochemistry at six different time points on the final day of TC2 (see arrowheads in Supplementary Fig. 5A). PER staining was performed on dissected brains and the number of PER^+^ neurons was determined (Fig. 5a, Supplementary Fig. 5B).

The most prominent differences in PER expression between wild-type and *pyx*^*3*^ mutant flies were observed in the PDF^+^ s-LNv and DN1 (Fig. 5a and Supplementary Fig. 5B). The number of PER-positive (PER^+^) DN1 neurons in *pyx*^*3*^ flies was significantly reduced during the entire cold phase (ZT14-ZT22), reinforcing the importance of the DN1 in temperature synchronization^3,28,30,39^. The number of PER^+^ s-LNv was similar between genotypes, but the timing of PER expression was different in the *pyx*^*3*^ mutant background. PER^+^ s-LNv’s peaked at ZT22 in wild-type flies, which was shifted to the opposite phase (ZT10) in *pyx*^*3*^ mutants (Fig. 5a). Likewise, the trough of PER expression was shifted from ZT6 to ZT22 in the *pyx*^*3*^ mutants (Fig. 5a). Interestingly, ZT10 and ZT22 during TC2 correspond to ZT23 and ZT11 of the initial light-dark cycle, reflecting peak and trough expression of PER in s-LNv during light-dark cycles^40^. Therefore a possible explanation for the massive shift in the phase of PER expression is that without PYX the s-LNv remains phase-locked to the initial light-dark cycle.

To validate these findings and to investigate whether the differences in PER expression could be rescued by the genomic *pyx* construct, we performed an independent experiment with wild-type, *pyx*^*3*^ and *pyxGE2;pyx*^*3*^ flies (Fig. 5b and Supplementary Fig. 5C). At ZT14 and ZT22 on the final day of TC2 (see arrowheads in Supplementary Fig. 5A) we observed the same differences in PER expression between genotypes in the DN1 and s-LNv as before (compare Fig. 5a, b). At ZT14, the number of *pyx*^*3*^ and wild-type PER^+^ s-LNv was again identical, whereas at ZT22 only two PER^+^ *pyx*^*3*^ s-LNV were detectable compared to 3–4 PER^+^ s-LNv in the wild-type and rescue flies (Fig. 5b). Similarly, the number of PER^+^ DN1 neurons was again significantly reduced at ZT14 and ZT22, but this was not (ZT14), or only partially restored (ZT22) by the genomic rescue construct. The lack of full DN1 rescue resembles the partial rescue of behavioral resetting (Fig. 1a) and again highlights the importance of the DN1 for temperature synchronization.

In the remaining clock neuronal groups PER expression did not vary significantly between *pyx*^*3*^ mutants and controls, or the differences were not consistent between experiments (Supplementary Fig. 5B, C). Taken together, the s-LNv and DN1 subsets are neuronal targets of *pyx*-dependent temperature input to the clock, although other neurons may also be affected. While in *pyx*^*3*^ PER oscillations in the s-LNv occur in opposite phase to the controls, the numbers of PER-expressing DN1 are constitutively reduced during the entire cold phase.

To independently determine the effects *pyx*^*3*^ on molecular synchronization, we applied a PER-luciferase transgenic strain (*8.0-luc*), which is expressed in subsets of the dorsally located clock neurons (in 16 °C:25 °C temperature cycle: 4 LNd, 5 DN1p, 2 DN2, plus a few DN3) but not in the LNv and peripheral clock cells^32,41^. Measuring bioluminescence emanating from *8.0-luc* flies is therefore a reliable method to estimate PER expression in dorsal brain clock neurons with high temporal resolution (e.g., see reference^28^). We recorded bioluminescence from *8.0-luc; pyx*^*3*^ and *8.0-luc; pyx*^*3*^/+ flies during ramped 16 °C:20 °C temperature cycle in constant darkness for 4 days with 30 min time resolution (Fig. 5c). Both genotypes exhibited robust bioluminescence oscillations, although the homozygous *pyx*^*3*^ flies appear to cycle with lower amplitude (note the lower peaks and troughs in the detrended data, Fig. 5c). A lower amplitude of *8.0-luc* oscillations fits well with the lower amplitude of PER oscillations observed in the DN1 (Fig. 5a). After 4 days the temperature cycle was delayed by 6 h and bioluminescence was measured for another 2 days during temperature cycles before flies were being exposed for 3 days to constant 16 °C in DD. Strikingly, while the *pyx*^*3*^/+ control flies immediately delayed their PER-LUC oscillations, the homozygous *pyx*^*3*^ mutants continued oscillating with their original phase (Fig. 5c). We replotted both the raw and detrended data of the 3 days around the temperature cycle shift showing that after the shift luminescence levels of the control flies continue to decrease, while that of the mutant flies increases (Fig. 5c, black double arrows). Also, in the final constant conditions control flies keep oscillating with high amplitude, while oscillations in *pyx*^*3*^ flies show a drastic reduction in amplitude (Fig. 5c). We performed the same experiment with shifted 16 °C:25 °C temperature cycle and although the *8.0-luc; pyx*^*3*^ flies delayed their oscillations after the 6 h shift, the delay was smaller compared to the control flies (Supplementary Fig. 6). Also, as in the 16 °C:20 °C temperature cycle, the amplitude of *8.0-luc* oscillations was drastically reduced in constant conditions compared to the controls (Supplementary Fig. 6).

### Synchronized period expression in the s-LNv maintains temperature-synchronized behavior

The lack of PYX causes abnormal behavior during temperature cycles, which leads to an altered activity phase in subsequent constant conditions (Figs. 1, 2). This indicates that the molecular oscillations that underlie behavioral rhythms in constant conditions are also altered in *pyx*^*3*^ mutants. The data obtained with the *8.0-luc* reporter already showed that PYX is required for maintaining robust PER-LUC oscillations in constant conditions in dorsal clock neurons (Fig. 5c and Supplementary Fig. 6). To see if the changes in the phase of PER expression in the s-LNv observed in *pyx*^*3*^ flies under temperature cycle conditions (Fig. 5) are also maintained, we performed PER stainings at three different time points on the 1st day of constant conditions (see arrowheads in Supplementary Fig. 5B). To ensure that the flies were exposed to the same conditions as those in Fig. 1, and to determine if the activity peak differences in constant conditions are present on the 1st day of constant conditions (those plotted in Fig. 2 were determined on the second and third day), we recorded the behavior of a small cohort of flies that were kept in the same incubator as those collected for staining (Supplementary Fig. 7). The timing of the behavioral peak during the first day of constant-running conditions was quantified using circular phase plots. This behavioral analysis resulted in an average phase plot (Supplementary Fig. 7A) and single fly phase plots that were overlaid on the average actograms depicting fly activity on day 1 of constant conditions of the same flies (Supplementary Fig. 7B). These control data show the expected phase difference between *pyx*^*3*^ and wild-type flies and absolute phase values similar to those calculated for days 2 and 3, whereas the rescue flies behaved similar to wild-type (Supplementary Fig. 7A, B). Thus, we are confident that the flies collected for staining also show the behavioral differences caused by the absence or presence of PYX function.

On the 1st day of free run, the number of PER^+^ neurons in wild-type flies peaked at CT22 identical to the result during temperature cycles (compare Supplementary Fig. 7C to 5A, B). *pyx*^*3*^ neurons also show identical PER kinetics during free-running conditions and temperature cycles (compare Supplementary Fig. 7C to 5A, B): at CT14 the number of PER^+^ s-LNv is not different from wild-type, but is significantly reduced at CT22 (Supplementary Fig. 7C). Finally and importantly, the rescue flies again show a high number of PER^+^ s-LNv at CT22, indicating rescue of the s-LNv phenotype of *pyx*^*3*^ flies, corresponding with the behavioral rescue under constant conditions. The number of PER^+^ DN1 during the subjective cold phase was reduced in *pyx*^*3*^ mutants compared to wild-type similar to what we observed during temperature cycle (compare Supplementary Fig. 7C with 5A, B). But this reduction was also observed in the rescue flies, indicating that the phase differences between *pyx*^*3*^ mutants and wild-type flies in constant conditions are caused by differences in PER expression within the s-LNv.

## Discussion

We report here a surprising function of the TRP channel PYX and the antennae for temperature synchronization of the circadian clock. Based on previous work^3,13,18,29^ we predicted PYX-mediated temperature sensation within the cap cells of ChO to be relevant for temperature resetting. Although it is likely that PYX in these and in other peripheral nervous system cells also contributes to temperature synchronization, the fact that we could partially rescue synchronization in *pyx* mutants by removing their antennae suggests a novel function for PYX within the antennae. Although it is formally possible that PYX functions outside the antennae to gate thermal antennal input, the fact that PYX is not expressed in the central nervous system indicates that this TRPA channel functions within antennae and other sensory organs to mediate temperature synchronization of the circadian clock. The antennae are not required for temperature synchronization^3,13^, so how can their removal restore behavior of *pyx* mutants (Fig. 1b)? PYX presumably is preferentially permeable for potassium ions, which can explain its putative protective role from inappropriate neuronal firing during high temperatures^19^. Because of the high intracellular potassium concentration at rest, PYX activation will result in potassium efflux, thereby hyperpolarizing the cell and effectively inhibiting neuronal activity. Our results fit with this idea, because *pyx* mutants exhibit higher calcium levels during temperature cycles (Fig. 3), and constitutive activation of *pyx* expressing cells mimics the *pyx* mutant phenotype (Fig. 4). Because the increased calcium levels are specific for temperature-cycling conditions, we postulate that PYX normally gates temperature input from antennal PYX neurons to the clock neurons. Good candidates are the thermosensitive neurons in the fly aristae^30,31^, which may be at the receiving end of a neuronal circuit connecting antennal *pyx* neurons to the circadian clock described recently^21,42^. Interestingly, we were able to detect *pyx–gal4* signals at the base of the antennae, but if these or other antennal *pyx* expressing cells (Supplementary Fig. 1) contribute to temperature synchronization is still open. Nevertheless, antennal *pyx* neurons project to anterior cell neurons in the brain^21^, which send presynaptic projections to the dorsal protocerebrum, where they contact s-LNv projections^42^. In addition anterior cell neurons directly contact the DN1 to mediate ‘prolonged morning wakefulness’’ at temperatures > 30 °C^43^. Interestingly, the s-LNv and the DN1 of *pyx*^3^ mutants are among the clock neuronal subgroups in which the phase of PER oscillations or the number of PER expressing cells was reproducibly and significantly altered compared to wild-type (Fig. 5 and Supplementary Figs. 5B, C and 7), both during temperature cycles and in constant conditions. Because of the limited time point analysis in constant conditions, we cannot distinguish if PER expression in the s-LNv of *pyx*^*3*^ flies is rhythmic or not (Supplementary Fig. 7). However, the observed behavioral rhythmicity in constant conditions (Fig. 1a) suggests that rhythmic PER expression is not abolished in *pyx*^*3*^ flies. In addition, using a PER-LUC fly line (*8.0-luc*) reporting PER expression in subsets of the LNd and DN, we could show that PYX is required for molecular synchronization to temperature cycles and maintenance of rhythmicity during constant conditions (Fig. 5c and Supplementary Fig. 6). In summary, we propose that in the absence of PYX inappropriate signals from antennal *pyx* neurons reach the s-LNv and DN1 via the anterior cell neurons, resulting in altered PER oscillations and levels during temperature cycles.

Antennal removal also improves synchronization to cycles of vibration and silence^29^. Synchronization by vibration and silence shares several commonalities with temperature synchronization: The flies’ activity profile during and after exposure to both synchronization cycles is very similar and distinct from synchronization by light:dark cycles, and both rely on ChO function^29^. To achieve synchronization by vibration and silence, the artificial two-frequency vibration applied, most likely activated only subsets of the body and antennal ChO, and removal of the antennae may therefore have eliminated an inappropriate signal to the clock neurons, similar as described above for *pyx* mutants. Together, the two independent examples of improved synchronization after antennal ablation strongly indicate a connection from antennal to clock neurons, which depending on the conditions can either promote or disturb clock synchronization.

Removal of the antennae only partially rescues synchronization of *pyx* mutants. This could mean that PYX does indeed act in other peripheral temperature sensors (e.g., the femur ChO cap cells) to sense or signal temperature information to the brain clock. Alternatively, PYX may function to gate temperature signals perceived by other thermal receptors in the fly peripheral nervous system. In this regard, *pyx* is expressed widely within the adult peripheral nervous system, including bristle, maxillary palp, and proboscis neurons^19^, some of which may contain unknown thermoreceptors.

Like PYX, the TRP channel TRPA1 functions over a wide temperature range^44^. For example, while at high temperatures above 25 °C TRPA1 activation is presumably direct^45^, activation at lower temperatures (18 °C) is mediated by a G-protein coupled transduction cascade involving Rhodopsin 1, Gq, and PLC-ß^46,47^. In addition, TRPA1 also plays nonthermal roles in chemical sensing (e.g., see references^48,49^). It is therefore likely that at lower temperatures (16–25 °C, this study; 27 °C^21^) PYX activation is also indirect. In general, recent studies indicate that sensory proteins are less specific than previously thought, presumably even able to detect different sensory modalities, like for example light and temperature reception reported for rhodopsins^50^. In the case of TRP channels this means a certain channel can act both as sensory protein (e.g., TRPA1 and PYX at their high direct activation temperatures) or as gated channels, responding to a sensory transduction cascade (e.g., TRPA1 at 18 °C, see reference^46^).

## Methods

### Fly strains

Fly strains were reared on standard yeast-containing fly food and were entrained to a 12:12 h light-dark cycle at constant 25 °C and 60% humidity. All fly strains have been described previously: *pyx*^*3*^ and *pyx*^*GE2*^;*pyx*^*3*^ ^19,22^, *UAS-pyx RNAi*;^19,22^ *UAS-NaChBac*^38^, *UAS-mCD4-tdTomato* (BL35841), *actin-gal4* (BL4414); *pyx–gal4*;^17^ *tim-gal4*^51^*, R18H11-lexA*^52^*, Clk4.1-lexA*^53^, *cry-lexA* (gift from Francois Rouyer), *UAS-TRIC* (BL62830)*, lexA-op-luc*^52^*, 8.0-luc*^41^, *repo-lexA::GAD-lexAop-mCD8GFP*^54^. The *pyrexia (pyx)* null-allele *pyx*^*3*^ was generated by imprecise excision of a P-element, which resulted in lack of both PYX isoforms^19^. *pyx*^*GE2*^;*pyx*^*3*^ flies carry a genomic rescue construct as well as the *pyx*^*3*^ allele^19^. Both lines carried the *s-tim* allele^55^, therefore *y w; s-tim* flies were used as a control in all experiments performed (referred to as wild-type).

### Locomotor activity recording and analysis

Locomotor activity recording was performed as described previously^9,18^. Approximately 3-day-old male flies were loaded in glass tubes that contained food (2% agarose and 4% sucrose) on one side. Tubes were plugged with cotton wool and placed in *Drosophila* activity monitors (DAM2 system, TriKinetics, Waltham, MA) to record locomotor activity during the experiment. Environmental conditions were programmed in light and temperature controlled incubators (Percival). Light dark and temperature cycle conditions were always programmed as 12:12 h rectangular cycles. Light conditions changed immediately whilst temperature inside the incubator reached the new set point within 30 min temperature cycles were always in constant darkness. By placing a water bucket inside the incubator during the experiments humidity was kept between 60% and 90%. Flies were exposed to 3 days of light-dark cycles at 20 °C, after which the lights stayed off for the remainder of the experiment. Light-dark cycles were followed by 6 h of darkness 20 °C, followed by 5 days of constant darkness temperature cycle 20 °C:16 °C (which was thus effectively delayed by 6 h compared to light-dark). After 5 days, the last cold phase was extended by 7 h of darkness at 16 °C, followed by 5 days of constant darkness temperature cycle 20 °C:16 °C (thus effectively delayed by 7 h compared to the previous temperature cycle). After the second temperature cycle (TC2) flies were released into free-running conditions with a constant temperature of 20 °C (constant darkness 20 °C). Activity data were recorded every minute and were analyzed as total activity counts per 30 min actograms of locomotor activity data were generated in Matlab, using the Fly toolbox^33^. After visual inspection of individual actograms, the flies that did not survive until the end of the experiment were excluded from analysis. Histograms were generated in Microsoft Excel and showed the average activity of the last 3 days in TC2 (days 3–5) or the second and third day in free-running conditions (DD2–DD3). Phases of individual flies during free-running conditions were quantified using Matlab and the Fly toolbox as previously described^33,56,57^. Briefly, activity data were smoothed using a low-pass Butterworth filter, set to remove periodicities <16 h, and the phase of the activity peak for each individual fly was calculated (average of DD2 and DD3 in Figs. 1c and 3b, Supplementary Fig. 1B, and DD1 in Supplementary Fig. 5A). Subsequently, two average vectors were compared to test for statistical differences between genotypes. Double plots showing distribution of individual absolute phase values were plotted using prism (GraphPad).

### Immunocytochemistry experiments and image processing

Wild-type, *pyx*^*3*^ and *pyx*^*GE2*^*;pyx*^*3*^ flies were first subjected to temperature cycles, after which PER expression in the brain was measured. Temperature cycles were similar to the behavioral experiments described above, except that TC2 was delayed with 6 (not 7) h compared to the previous TC1 and free-running conditions were at 16 °C (not 20 °C). Flies were collected for immunocytochemistry on the final day of TC2 (Fig. 5 and Supplementary Fig. 5A, B) or the first day of constant darkness (Supplementary Fig. 5C). Before collecting flies for immunocytochemistry, their locomotor behavior was analyzed. Flies that showed clear wild-type (in case of wild-type and *pyx*^*GE2*^*;pyx*^*3*^) and mutant (in case of *pyx*^*3*^) phenotypes were selected for dissection. For the data shown in Fig. 4a, flies were collected for immunocytochemistry at two different time points so that three independent experiments covered all six time points (experiment 1: ZT2 and ZT14, experiment 2: ZT6 and ZT18, and experiment 3: ZT10 and ZT22). The data shown in Fig. 5b are from an additional independent experiment (experiment 4: ZT14 and ZT22). For the data shown in Supplementary Fig. 5C, flies were collected for immunocytochemistry at three different time points within one experiment. At selected time points, flies were fixed in 4% paraformaldehyde in 0.1% PBS-T (phosphate-buffer saline supplemented with Triton X-100) for 11 h at 4 °C or 2.5 h at room temperature (RT). Flies were washed with 0.1% PBS-T for 3 × 5 min and 3 x 15 min at RT. Brains were dissected in PBS and subsequently blocked with 5% normal goat serum (NGS) in 0.5% PBS-T overnight at 4 °C. Samples were incubated with preabsorbed polyclonal rabbit anti-PER^58^ diluted 1:1000 and monoclonal mouse anti-PDF C7 (DSHB) diluted 1:500 and 5% NGS in 0.5% PBS-T for 48 h at 4 °C. After washing with 0.5% PBS-T, 3 × 5 min and 5 × 15 min at RT, samples were incubated with alexaFluor 488 conjugated goat anti-rabbit IgG diluted 1:500 and alexaFluor 647 conjugated goat anti-mouse IgG diluted 1:1000 (both Thermo Fisher Scientific) and 5% NGS in 0.5% PBS-T for 48 h at 4 °C. After washing with 0.5% PBS-T, 3 × 5 min and 4 × 15 min at RT, samples were mounted in Citifluor medium and stored at 4 °C.

For quantification of PER staining, confocal stacks of single brains were taken using a Leica SP8 laser scanning confocal microscope. Image acquisition settings were kept constant across all genotypes and time points within one experiment. For each hemisphere, the number of PER^+^ cells within each neuronal group was counted. For wild-type flies, the maximum number of cells for the different subgroups in DDTC 20 °C:16 °C was as follows: 12 for DN1, 2 for DN2, 4 for l-LNv, 4 for s-LNv, 1 for 5th s-LNv and 6 for LNd. No (or only very weak) PER staining was observed in DN3 and LPN subgroups under these conditions. For the image shown in Supplementary Fig. 1, *pyx–gal4* (chromosome *2*) flies were crossed to *UAS-mCD4-tdTomato/CyO; repo-lexA::GAD, lexAop-mCD8GFP/TM6* flies. Antennae from nonbalancer F1 offspring were dissected in 0.1% PBS-T and imaged without fixation using a confocal microscope (see above).

### Bioluminescence recordings

To determine intracellular calcium levels in *pyx–gal4* expressing cells the transcriptional calcium reporter TRIC (Transcriptional reporter of intracellular calcium) was applied^34,35^. In this calmodulin-based system the LexA transcription factor is reconstituted in the presence of high Ca^2+^ and activates luciferase expression (see Experimental Procedures). Due to the short half-life of luciferase activity in flies (4.5 h, see reference^36^) bioluminescence levels therefore reflect the current intracellular Ca^2+^ concentration. Expression of the TRIC-luciferase reporter genes within *pyrexia* expressing cells (using *pyx–gal4*) should therefore allow to compare Ca^2+^ levels between wild-type and *pyx*-mutant flies. For the experiments shown in Fig. 2 *w; lexA-op-luciferase; pyx*^*3*^ *pyx–gal4* flies were crossed to either *w; UAS-TRIC;* + (to obtain the *pyx*^*3*^/+ control flies) or to *w; UAS-TRIC; pyx*^*3*^ (to obtain the *pyx*^*3*^*/ pyx*^*3*^ mutant flies). For the experiments shown in Supplementary Fig. 3B the indicated *lexA* driver lines were crossed to *lexA-op-luciferase*. To quantify PER-LUC expression in dorsal clock neurons the *8.0-luc* transgenic was crossed into the background of the *pyx*^*3*^ mutant and *8.0-luc; pyx*^*3*^*/pyx*^*3*^ and *8.0-luc; pyx*^3^*/*+ flies were tested. 3–4-day-old male progeny from these crosses and stocks were transferred individually to wells of 96-well microtiter plates containing 100 μl of 5% sucrose, 1% agar, and 15 mM luciferin. Bioluminescence was detected with a TopCount Multiplate Reader (PerkinElmer) for 5 days during the temperature cycle, light-dark, or constant conditions as previously described (e.g., see reference^28^). Data were plotted using BRASS^59^. To determine the basic trend in the TRIC data (plotted as insets in Fig. 3a, c) and to generate the detrended *8.0-luc* data (Fig. 5c and Supplementary Fig. 6, right columns) we calculated the moving average for 24 h windows separated by the 30 min (or 1 h for Supplementary Fig. 6) sampling time using the ChronoStar program^37^.

### Statistics

Data are presented as mean ± S.E.M. Statistical tests were performed as described in the figure legends using GraphPad Prism software. The significance threshold was set as *p* < 0.05.

### Reporting summary

Further information on research design is available in the Nature Research Reporting Summary linked to this article.

## Supplementary information

Supplementary Information

Description of Supplementary Data

Supplementary Data 1

Supplementary Data 2

Supplementary Data 3

Supplementary Data 4

Reporting Summary

## Data Availability

All original data are available from the authors upon request. Raw data for Figs. 1–5 are deposited as Supplemental Data.
