# Supplementary material for: Temperature synchronization of the Drosophila circadian clock protein PERIOD is controlled by the TRPA channel PYREXIA
Source: Commun Biol. 2019 Jul 1;2:246. doi: 10.1038/s42003-019-0497-0 (PMC6602953; doi:10.1038/s42003-019-0497-0)

Supplementary Figure 1: *pyx-ga4* expressing cells in the antennae do not overlap with glia cells. Dual labelling of *pyx-gal4 > UAS-cd4TdTomato* and *repo-LexA > LexAop-mCD8Gfp* in the antennae. The repo driver labels glial cells (green). Left row: overlay, middle: *pyx-gal4*, right: *repo-LexA*). A) entire antenna. No co-labelling was observed between the *pyx-gal4* and *repo-LexA* expressing cells. Grey: bright field image. Scale bar: 50µm. B) Representative image of the 3rd segment. The glial projections are surrounding projections from *pyx*-expressing cells. Scale bar: 20 µm C) Representative image of the basal area of the arista. Glial projections are surrounding projections of *pyx*-expressing cells. Scale bar: 10 µm.

Supplementary Figure 1

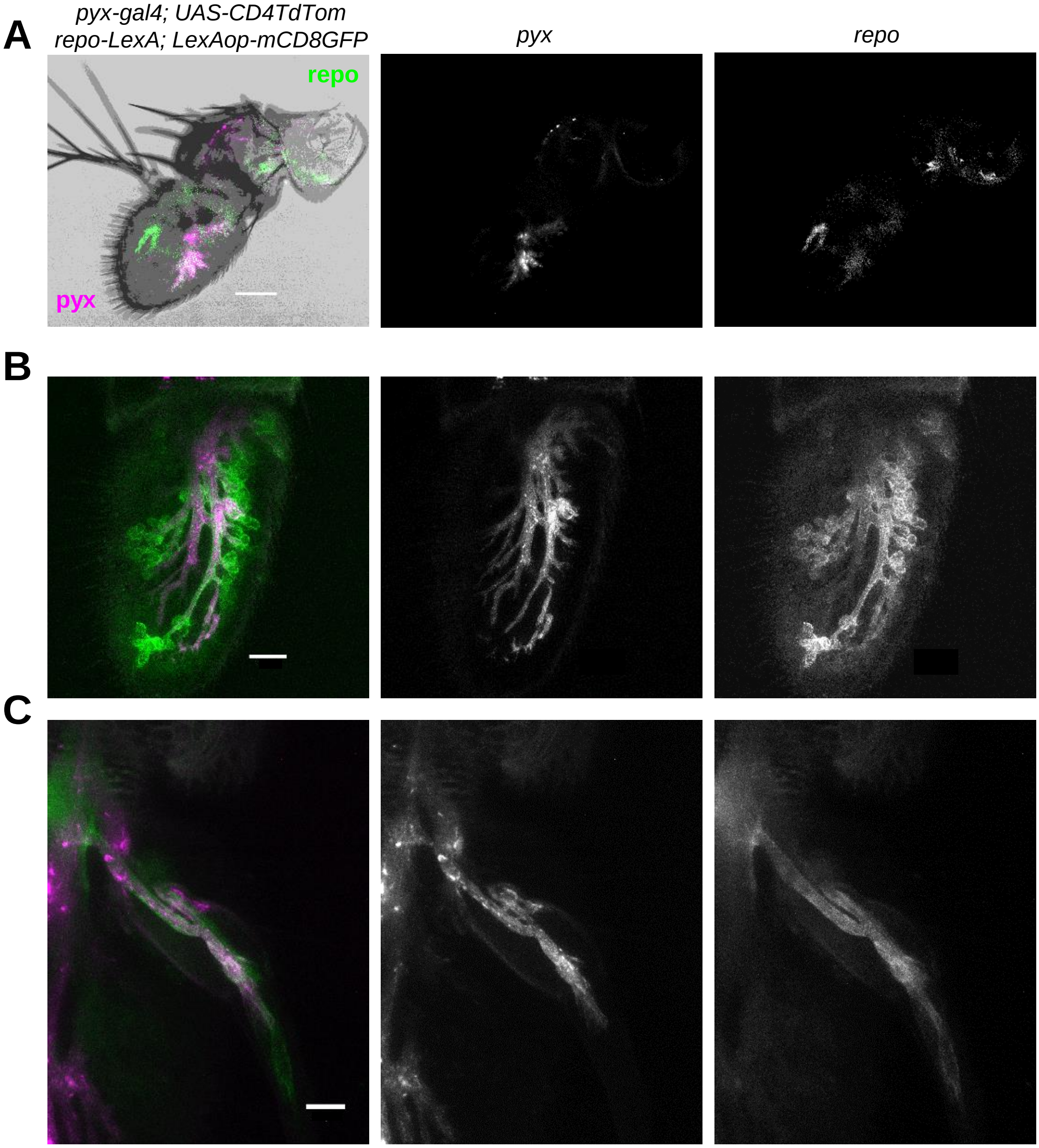

Supplementary Figure 2: The *pyx-gal4* driver is expressed in PYX expressing cells involved in temperature synchronization. PYX was knocked down by expressing the *pyx* RNAi construct (*UAS-pyx RNAi*) in different tissues (ubiquitous with *actin-gal4*; in *pyx* expressing cells with *pyx-gal4*; and in all clock cells with *tim-gal4*) and locomotor activity of these flies as well as the '*gal4*-only' and '*UAS-pyx RNAi*-only' controls was measured during and after DDTC 20°C : 16°C. For detailed experimental conditions, generation of actograms and histograms see legend to Figure 1A. Blue arrows point to activity peaks corresponding to the main activity of wild-type flies during the beginning of the warm phase in TC2 and subjective warm phase in subsequent constant conditions. Yellow arrow point to pyx^3-like activity in the cold phase during TC2 and subjective cold phase during constant conditions. Although the position of the main activity peak slightly varies between the control flies (blue arrows), main activity never coincides with the temperature increase as in pyx^3, or *pyx > pyxRNAi* and *actin > pyxRNAi* flies. Also, only the three latter genotypes exhibit activity peaks in the cold, or subjective cold phase (yellow arrows). For these reasons we don't think that the slight and non-qualitative differences between the control flies influence the interpretation of our results. For phase quantification see Supplementary Figure 3.

Supplementary Figure 2

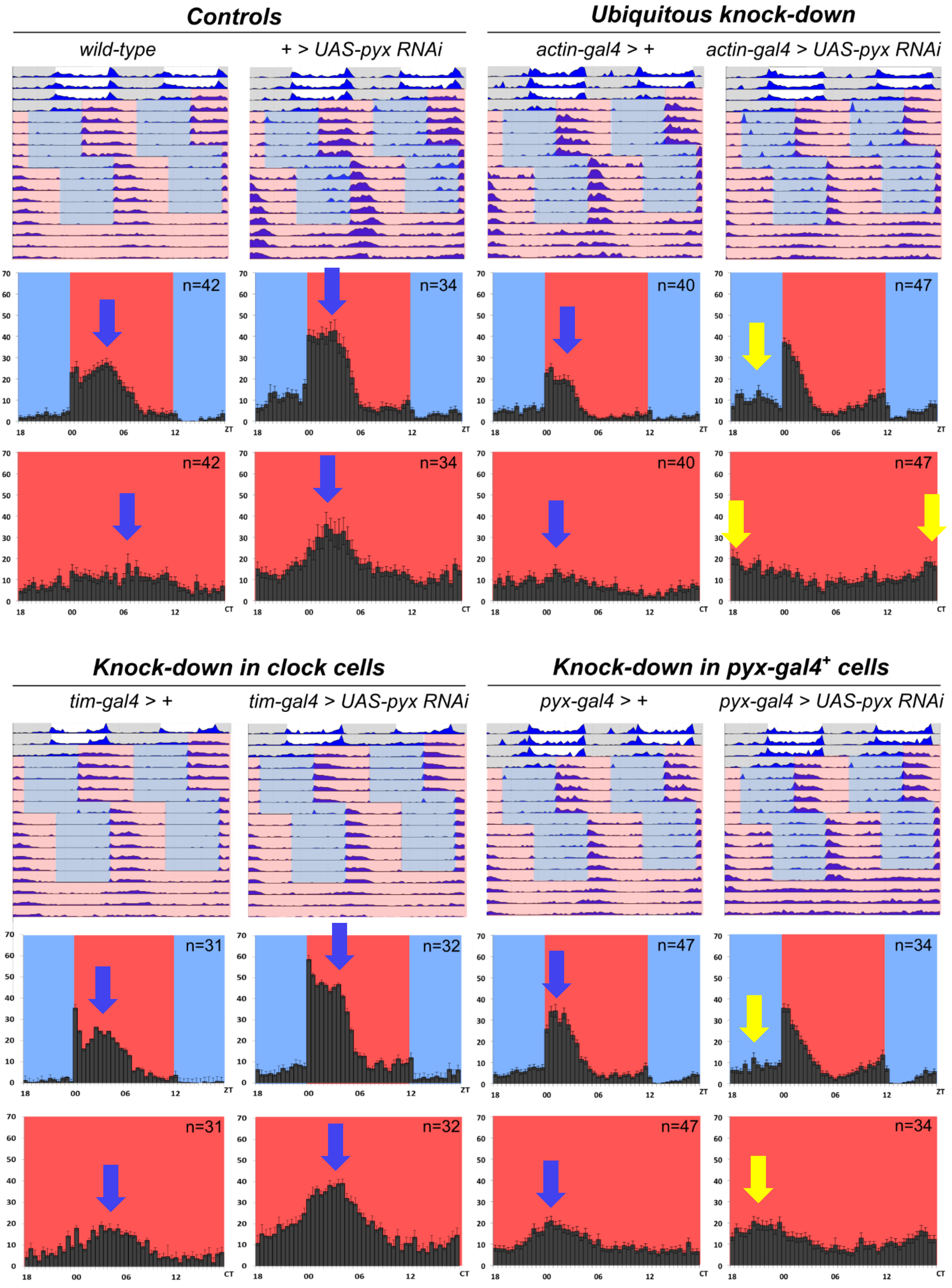

Supplementary Figure 3: Quantification of activity peak phase in flies expressing *pyx-RNAi* and controls A) Circular phase plots of a representative experiment showing the phase of peak activity during free-running conditions (DD 20°C, 2nd and 3rd day) after DDTC 20°C : 16°C. Dots represent individual flies. The direction of the vector indicates mean phase of a genotype and the magnitude of the vector indicates the coherence of the group (variance around the mean of one genotype). For the plots shown here coherence was not significantly different between genotypes. Genotypes are color-coded: wild-type is shown in bright blue, control genotypes are shown in black and experimental genotypes are shown in magenta: wild-type n=15, phase=4.7; *+>UAS-pyxRNAi* n=9, phase=3.0; *actin-gal4>UAS-pyxRNAi* n=16, phase=-5.9; *pyx-gal4>UAS-pyxRNAi* n=12, phase=-5.2; *tim-gal4>UAS-pyxRNAi* n=16, phase=-0.7; *actin-gal4>+* n=15, phase=1.6; *pyx-gal4>+* n=14, phase=1.1; *tim-gal4>+* n=14, phase 4.9). The difference of peak activity (in hr) of the different genotypes compared to that of the respective control is given below the phase plots (Dif with associated p values) **B)** Double plot of peak phase during free-running conditions (DD 20°C) after DDTC 20°C : 16°C (2nd and 3rd day), combining three experiments. Genotypes are color-coded: wild-type is shown in bright blue, control genotypes are shown in grey and experimental genotypes are shown in magenta (wild-type n=70, *+>UAS-pyx RNAi* n=34, *actin-gal4>+* n=40, *actin-gal4>UAS-pyx RNAi* n=47, *pyx-gal4>+* n=46, *pyx-gal4>UAS-pyx RNAi* n=33, *tim-gal4>+* n=31; *tim-gal4>UAS-pyx RNAi* n=32).

Supplementary Figure 3

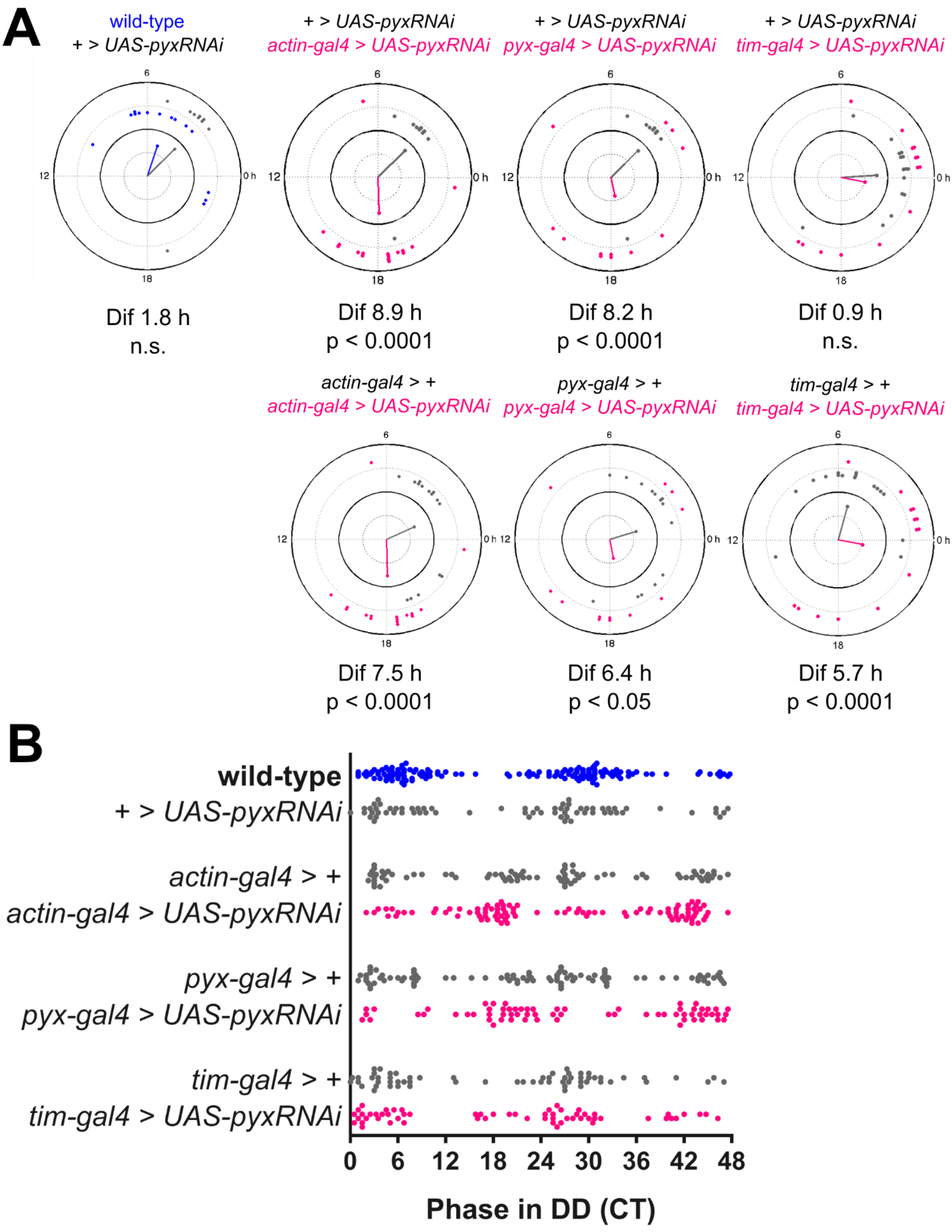

Supplementary Figure 4: Pyrexia also affects calcium levels during lower temperatures and temperature cycles induce rhythmic LexA-driven luciferase activity. A) TRIC calcium measurements as described in Figure 3 during (n=13 for *pyx*^{3} and n=8 for *pyx*^{3}/+). Significance of differences between genotypes was determined by t-test (*p < 0.05). **B)** Bioluminescence recordings of male *R18H11-lexA/lexA-op-luc* flies exposed to ramped 16°C : 25° TC for 3 days, followed by 2 days at constant 16°C and kept in DD throughout the experiment. Bioluminescence (Counts Per Second, CPS) from individual flies kept on luciferin containing food was measured every 30 min using a TopCount plate reader as previously described [62]. Similar oscillations were observed after driving *lexA-op-luc* with *Clk4.1-lexA* (like R18H11 expressed in a subset of DN1 clock neurons: Cavanaugh et al 2014), and *cry-lexA* (expressed in cryptochrome expressing cells) during ramped 16°C : 25° TC for 5 days, indicating that LexA activity increases with temperature. Note the high bioluminescence counts in *R18H11-lexA* and *Clk4.1-lexA* flies, indicating that these drivers are expressed in many cells outside the brain and not just in subsets of the DN1. Inset in **(B)** and color coded bars below x-axis depict temperature ramping during TC, with blue and red indicating colder and warmer temperatures, respectively.

Supplementary Figure 4

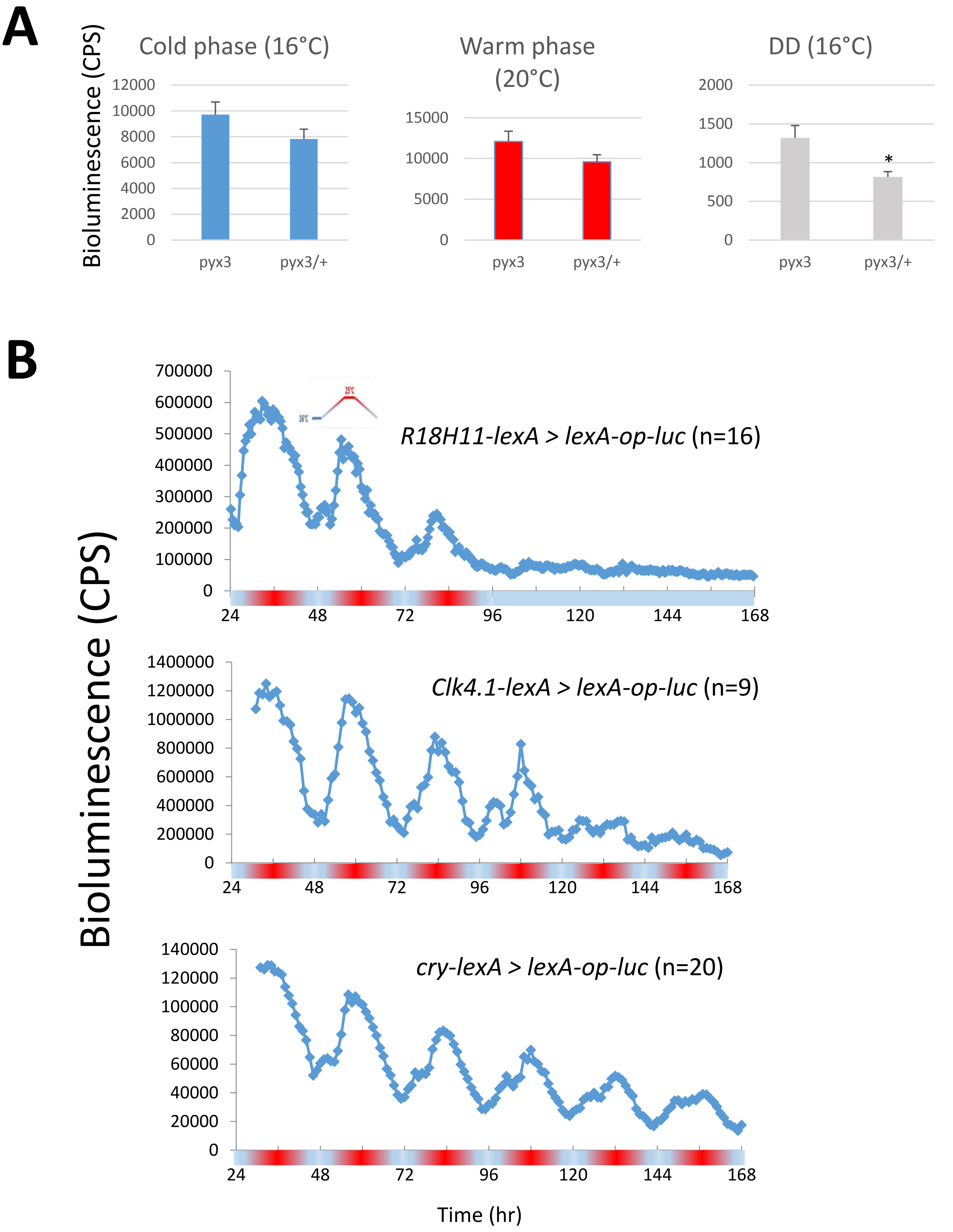

Supplementary Figure 5: PYX is not required for temperature synchronization of PER expression in most of the circadian clock neurons. A) Double plotted actograms show average locomotor activity of wild-type and *pyx*^{3} flies during DDTC 20°C : 16°C, before they were collected for immunocytochemistry on the final day of TC2 at 6 different time points (ZT2, ZT6, ZT10, ZT14, ZT18, ZT22), indicated with red arrowheads. Average activity is plotted in blue. 3 days of LD cycles (at constant 20°C) were followed by 5 days of 20°C : 16°C TCs (TC1) which were 6 hr delayed compared with previous LD, followed by 5 days of 20°C : 16°C TCs (TC2) which were 6 hr delayed compared with TC1. White and grey background colors indicate lights-on and lights-off respectively, red and blue background colors indicate warm (20°C) or cold (16°C) temperature respectively. TCs were always in constant darkness. n numbers wild-type n=10; *pyx*^{3} n=8. **B)** Average number of l-LNv, 5th s-LNv, LNd and DN2 PER$^+$ neurons detected at the 6 time points as indicated in **(A)**. Error bars represent s.e.m. n varies between 6 and 18 hemispheres per time point. 20°C (red) and 16°C (blue) TC is indicated with the bar below each graph. Significant differences in the number of PER$^+$ neurons between genotypes were determined using 2-way ANOVA followed by Sidak's multiple comparisons test in the case of significant interaction or significant effect of genotype (* $p<0.05$) (for all groups there was a significant genotype x time point interaction except for l-LNv. For l-LNv there was a significant effect of genotype alone, but multiple comparisons showed no significant differences per time point). Co-staining with PDF antibody allowed identification of PDF-negative 5th s-LNv, whereas other neurons were identified by position within the brain relative to the PDF-expressing cell bodies and their projections. **C)** Independent experiment analyzing number of PER$^+$ neurons at ZT14 and ZT22 on the final day of TC2 and also including *pyxGE2, pyx*^{3} rescue flies. Error bars represent s.e.m. n numbers range between 18 and 28 hemispheres per time point. Significant differences in the number of PER$^+$ neurons between genotypes were determined using 2-way ANOVA followed by Tukey's multiple comparisons test in the case of significant interaction or significant effect of genotype (different

letter means p<0.05 e.g. ‘a’ vs. ‘b’, shared letter means n.s. e.g. a vs. ab) (there was a significant genotype x time point interaction for LNd; there was no significant interaction but a significant effect of genotype alone for l-LNv; there was a significant effect of time point alone in 5th s-LNV hence no multiple comparisons of genotypes; there were no significant differences for DN2). LPN and DN3 were not analyzed, because PER expression was undetectable (LPN) or very weak (DN3) (cf. [32]).

Supplementary Figure 5

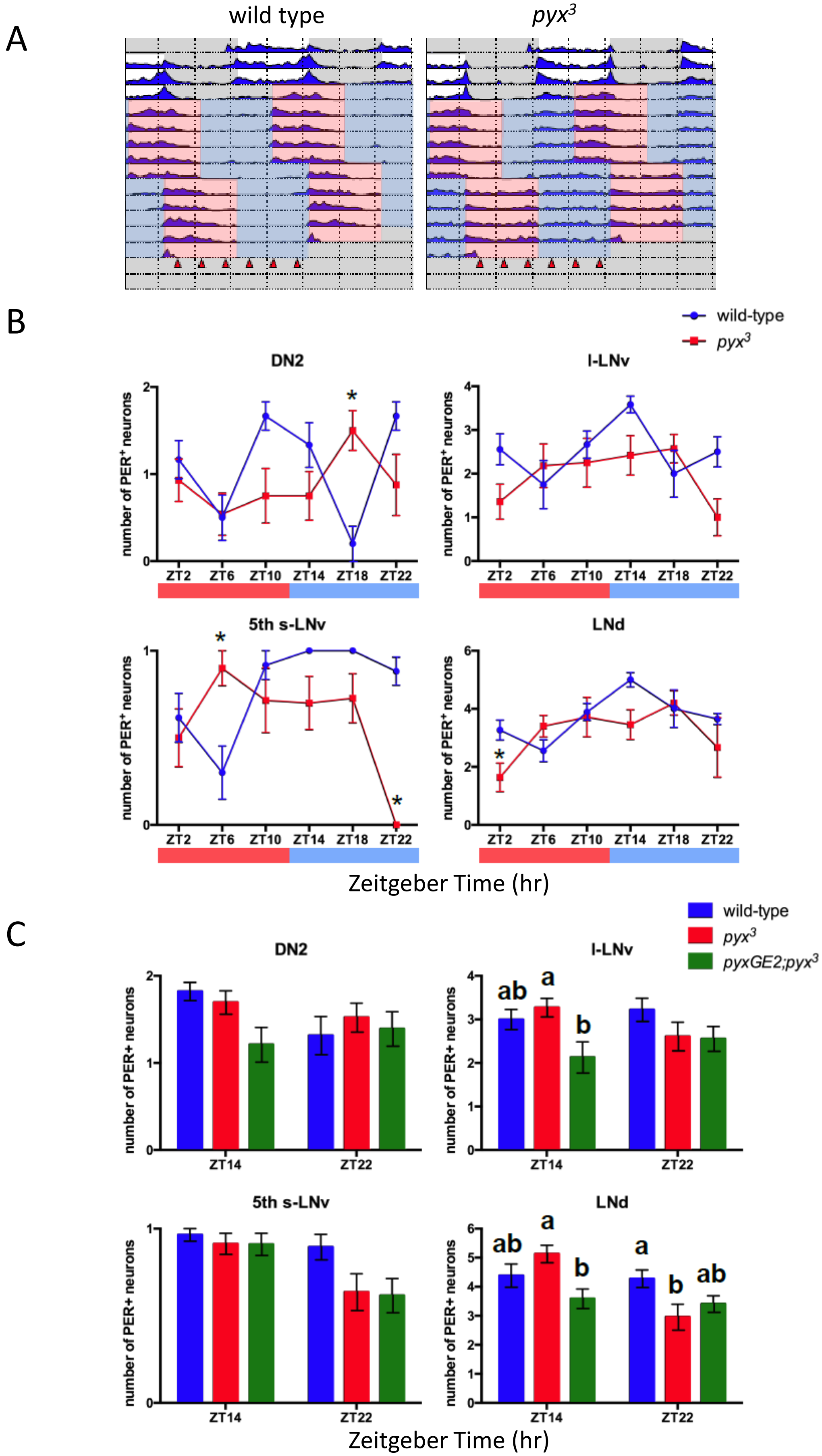

Supplementary Figure 6: Pyrexia also affects molecular synchronization to 16° C:25°C temperature cycles. Bioluminescence recordings of *pyx*^{3} and *pyx*^{3}/+ flies carrying the *8.0-luc* PER-LUC reporter. Flies were exposed to ramped 16°C : 25°C TC and bioluminescence was recorded every hour as described for Figure 5C. Delay of the TC is indicated by brackets and red arrows. Raw data are shown to the left, detrended data to the right (see Experimental Procedures). Day of the TC-shift and two subsequent days are shown in the bottom row. Arrows point to trough phase difference between mutants and controls after the TC-shift.

Supplementary Figure 6

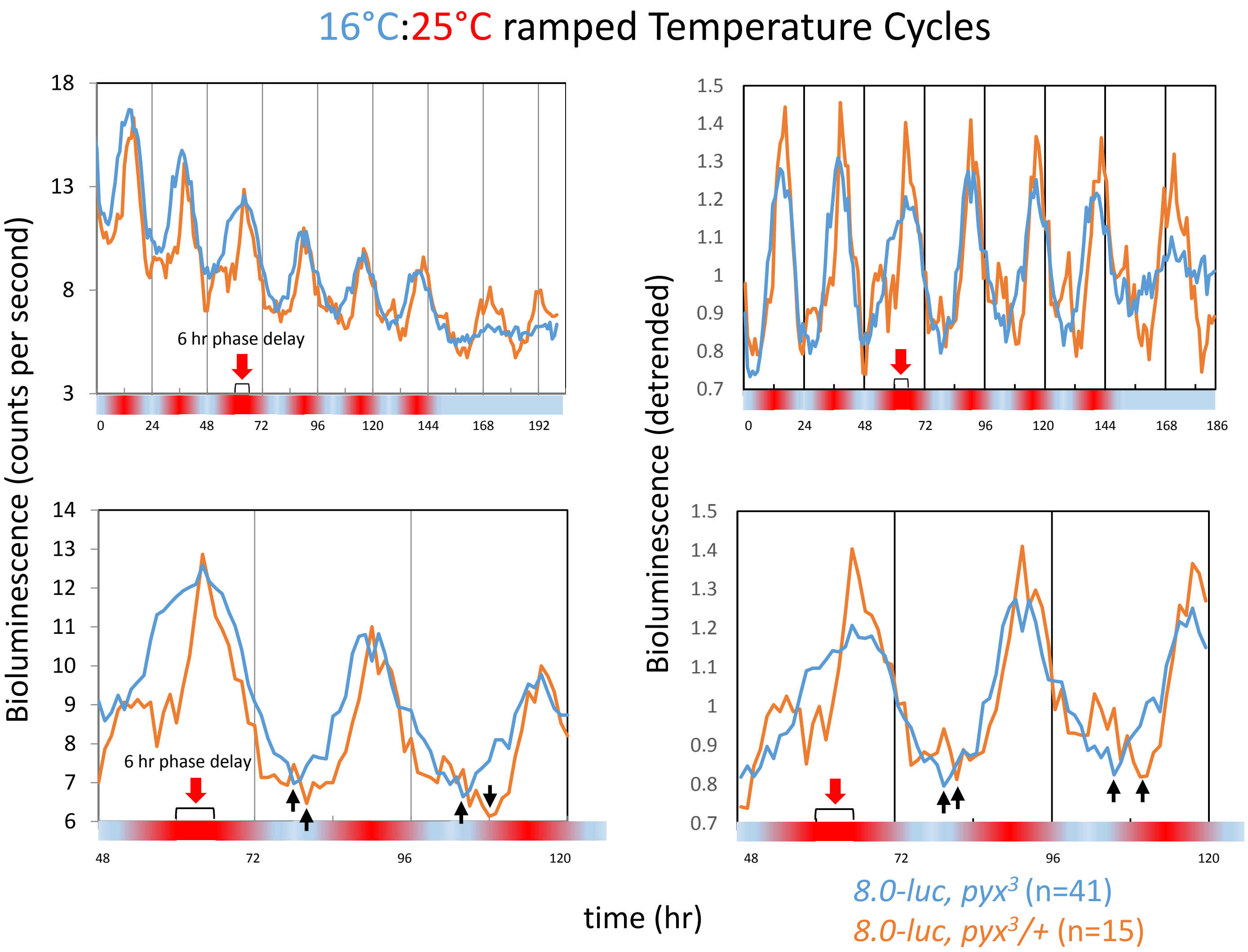

Supplementary Figure 7: Behavioral and molecular phenotypes of *pyx*^{3} mutants persist under constant conditions. A) Phase plot showing the difference of peak activity (in hr) of the genotypes indicated on the x-axis compared to that of wild-type (Δ phase). Δ phase from one experiment was determined on the 1st day of free-run after exposure to shifted TC (as depicted in Figure 1) using circular statistics [33] (*p < 0.05). Wild-type (n=9), *pyx*^{3} (n=4), *pyxGE2, pyx*^{3} (n=8). **B)** Single plot of peak phase during the first day of free-running conditions (DD 16°C) after DDTC 20°C : 16°C (same data as in **(A)**), overlaid on average actograms of the same flies, comparing wild-type, *pyx*^{3} and *pyxGE2;pyx*^{3} flies. Genotype color-coding and n numbers are as in **(A)**. Red arrowheads indicate collecting times for anti-PER staining shown in **(C)**. **C)** Average of PER$^+$ s-LNv at the indicated time points comparing wild-type (blue), *pyx*^{3} (red) and *pyxGE2;pyx*^{3} (green) flies on the first day of free-running conditions (DD 16°C) after DDTC 20°C : 16°C. Error bars represent s.e.m. n numbers range between 10 and 18 hemispheres per time point. Blue bar below the graph indicates constant 16°C. Significant differences between genotypes were determined using 2-way ANOVA followed by Tukey's multiple comparisons test. There was a significant genotype x time point interaction and significant differences between genotypes are indicated as follows: *(red) means significant difference between wild-type and pyx3. *(green) means significant difference between wild-type and *pyxGE2;pyx*^{3}. Significant differences between *pyx*^{3} and *pyxGE2;pyx*^{3} are not shown in the figure (* p<0.05).

Supplementary Figure 7

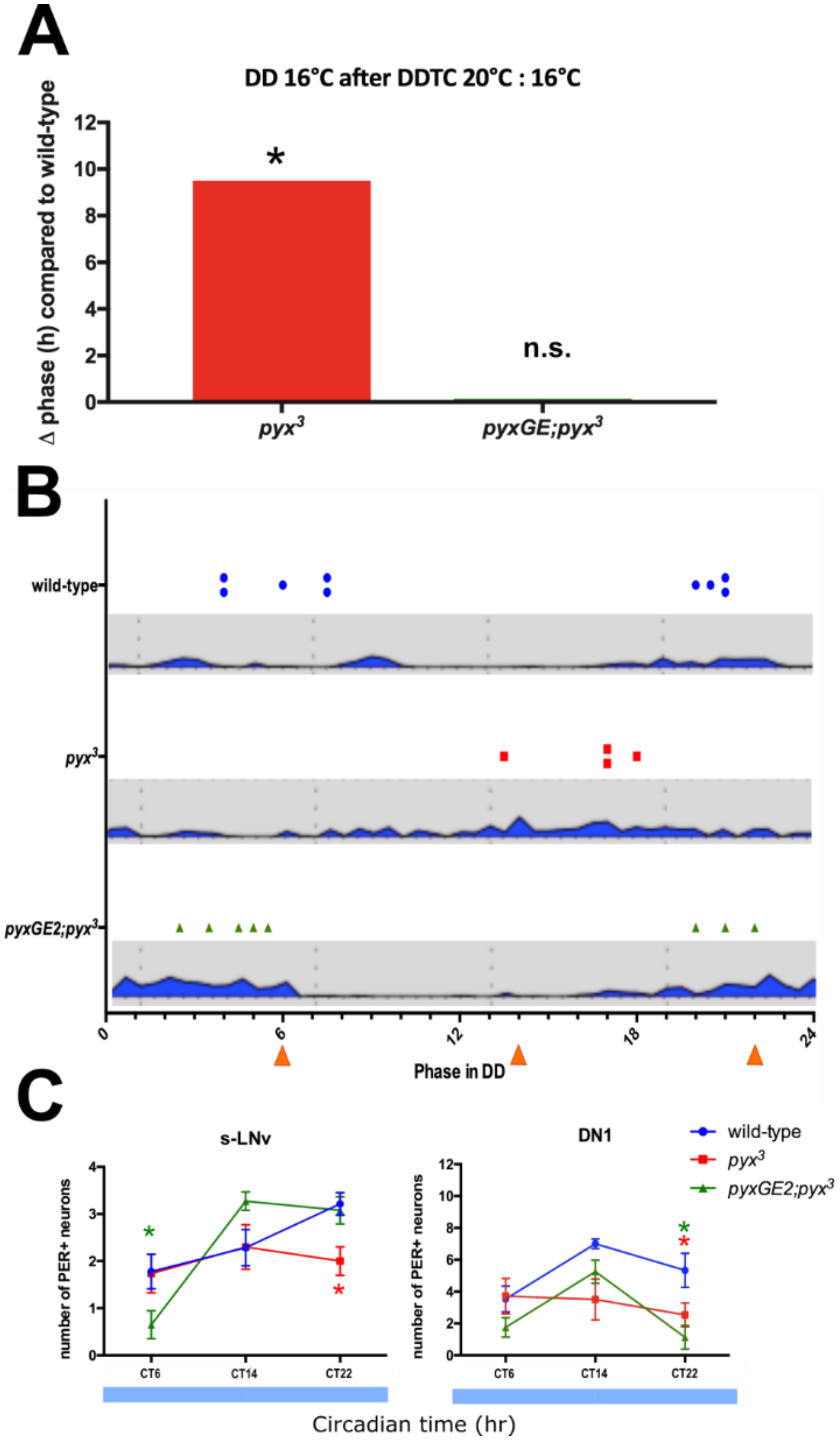

Supplement: Supplementary file 1 — Supplementary Information [file 42003_2019_497_MOESM1_ESM.pdf]
