# Supplementary material for: Temperature synchronization of the Drosophila circadian clock protein PERIOD is controlled by the TRPA channel PYREXIA
Source: Commun Biol. 2019 Jul 1;2:246. doi: 10.1038/s42003-019-0497-0 (PMC6602953; doi:10.1038/s42003-019-0497-0)
Supplement: Supplementary file 2 — Description of Supplementary Data [file 42003_2019_497_MOESM2_ESM.pdf]

Supplementary Data 1: Source data used to generate plots in Figure 1 and Figure 2

Supplementary Data 2: Source data used to generate plots in Figure 3

Supplementary Data 3: Source data used to generate plots in Figure 4

Supplementary Data 4: Source data used to generate plots in Figure 5
